# A versatile sample-delivery system for X-ray photoelectron spectroscopy of in-flight aerosols and free nanoparticles at MAX IV Laboratory

**DOI:** 10.1107/S1600577524005411

**Published:** 2024-08-07

**Authors:** C. Preger, J. Rissler, A. Kivimäki, A. C. Eriksson, N. Walsh

**Affiliations:** a Ergonomics and Aerosol Technology Lund University Box 118 221 00Lund Sweden; b MAX IV Laboratory Lund University Box 118 221 00Lund Sweden; c NanoLund Lund University Box 118 221 00Lund Sweden; d RISE Research Institutes of Sweden Scheelevägen 17 223 70Lund Sweden; University of Essex, United Kingdom

**Keywords:** aerosols, free nanoparticles, in-flight, sample-delivery systems, MAX IV, X-ray photoelectron spectroscopy

## Abstract

Aerosol science is of utmost importance for both climate and public health research, and in recent years X-ray techniques have proven effective tools for aerosol-particle characterization. To date, such methods have often involved the study of particles collected onto a substrate, but a high photon flux may cause radiation damage to such deposited particles and volatile components can potentially react with the surrounding environment after sampling. These and many other factors make studies on collected aerosol particles challenging. Therefore, a new aerosol sample-delivery system dedicated to X-ray photoelectron spectroscopy studies of aerosol particles and gas molecules in-flight has been developed at the MAX IV Laboratory. The aerosol particles are brought from atmospheric pressure to vacuum in a continuous flow, ensuring that the sample is constantly renewed, thus avoiding radiation damage, and allowing measurements on the true unsupported aerosol. At the same time, available gas molecules can be used for energy calibration and to study gas-particle partitioning. The design features of the aerosol sample-delivery system and important information on the operation procedures are described in detail here. Furthermore, to demonstrate the experimental range of the aerosol sample-delivery system, results from aerosol particles of different shape, size and composition are presented, including inorganic atmospheric aerosols, secondary organic aerosols and engineered nanoparticles.

## Abstract 2

A new mobile sample-delivery system dedicated to in-flight studies of pristine aerosols and unsupported nanoparticles using X-ray photoelectron spectroscopy at the MAX IV Laboratory in Lund, Sweden, is presented. The aerosol sample-delivery system brings an aerosol from atmospheric pressure to the experimental chamber in a continuous flow, and the design, operation and commissioning results of the system are presented.

## 1. Introduction

Aerosols influence climate, contributing to radiation forcing, and air pollution. They also contribute to viral transmission and play a role in the generation of new materials. An aerosol is defined as a two-phase system consisting of particles suspended in a gas, and generally exists under close to atmospheric conditions. Particles in aerosols range in size from a few nanometres to several micrometres, and have varying morphologies and compositions. Relevant aerosol dynamics and chemical reactions are known to occur whilst the particles are suspended in the gas environment, and it is therefore essential to have the possibility to analyze the particles in flight, in their aerosol phase.

Many aerosol-particle characterization methods rely on prior particle collection onto a substrate. For volatile components and for particles that react with the surrounding environment, both the elemental content and the chemical form of the elements in the particles will be perturbed compared with the case of a pristine aerosol. A high photon flux may also cause radiation damage to the particles, and this becomes a substantial problem when studying the surface structure of embedded particles using, for example, X-ray photoelectron spectroscopy (XPS). With in-flight measurements, these, and other factors such as charge build-up for nonconductive samples, can be avoided, since the particle stream is constantly renewed. Moreover, in-flight measurements are performed on the true unsupported aerosol with the particles existing in the aerosol environment, without any potential influence and interactions with a substrate. Another advantage of the in-flight method is that the binding-energy calibration can be performed with the constantly available gas molecules from the aerosol, or gas added to the chamber. Furthermore, in-flight measurements do not require long sample preparation and aerosol generation parameters can be varied with immediate response in the photoelectron spectrum.

Compared with aerosol particles collected on a substrate, where particle number density can be controlled by sampling time, free flying aerosol-particle beams are generally much more dilute. Advances in in-flight aerosol instrumentation with the developments of the aerodynamic lens (ADL) (Liu *et al.*, 1995*a* ▸,*b* ▸) and the aerosol mass spectrometer (Jayne *et al.*, 2000 ▸) have made it possible to study aerosols in-flight by focusing the aerosol particles to a collimated particle beam. In parallel, more powerful synchrotron radiation sources have been developed that offer sufficient photon flux to ionize even dilute samples. The combined advances in aerosol technology and synchrotron radiation sources have motivated the development of in-flight aerosol sample environments at large-scale facilities. Shu *et al.* (2006 ▸) coupled an aerosol apparatus comprising an ADL and differential pumping sections to a vacuum chamber to sample aerosol particles from atmospheric pressure in a continuous flow. Similar systems, but with smaller modifications, were designed later at other synchrotron and free-electron laser facilities (Lindblad *et al.*, 2013 ▸; Antonsson *et al.*, 2013 ▸; Bogan *et al.*, 2008 ▸; Hickstein *et al.*, 2014 ▸). The key feature of all of these systems is that they produce a continuous flow of aerosol particles through an interaction region via transmission through an ADL. Aerosols have been studied with these types of systems at various light sources using soft X-ray spectroscopy (Shu *et al.*, 2006 ▸; Lindblad *et al.*, 2013 ▸; Antonsson *et al.*, 2013 ▸), elastic light scattering (Bresch *et al.*, 2008 ▸) and X-ray diffraction imaging (Bogan *et al.*, 2008 ▸, 2010 ▸). ADL systems have also been coupled to ambient-pressure XPS (Mysak *et al.*, 2010 ▸), velocity map imaging with vacuum ultraviolet (VUV) (Goldmann *et al.*, 2015 ▸) and VUV photoelectron spectroscopy (Su *et al.*, 2015 ▸). XPS is a surface-sensitive technique and has been used with the above-mentioned systems to study a range of different aerosols including engineered aerosol nanoparticles (Sublemontier *et al.*, 2014 ▸; Danilović *et al.*, 2020 ▸; Milosavljević *et al.*, 2018 ▸; De Anda Villa *et al.*, 2019 ▸; Benkoula *et al.*, 2015 ▸), salt aerosols (Pelimanni *et al.*, 2022 ▸; Unger *et al.*, 2020 ▸; Patanen *et al.*, 2022 ▸; Abid *et al.*, 2021 ▸; Antonsson *et al.*, 2018 ▸, 2015 ▸) and soot particles (Ouf *et al.*, 2016 ▸).

Here we present a new sample-delivery system that has been developed at the MAX IV Laboratory to facilitate in-flight measurements on aerosol particles and free unsupported engineered nanoparticles (ENPs). The aerosol sample-delivery system (ASDS) has been designed such that it can be used at different beamlines at MAX IV, extending the available photon energy range and other photon properties to enable a wider variety of experiments. Furthermore, the ASDS is designed such that it allows modifications, based on the experimental application. In this article, the design and operation of the ASDS, as well as selected commissioning results for a range of different particle types, are presented.

## 2. Aerosol sample-delivery system

### 2.1. Design of the aerosol sample-delivery system

The design of the ASDS is based on the same principle as the sample-delivery systems found at BESSY II (Antonsson *et al.*, 2013 ▸), SOLEIL (Lindblad *et al.*, 2013 ▸) and ALS (Shu *et al.*, 2006 ▸). The design makes it possible to mount the ASDS in different configurations depending on experimental requirements but has so far only been used at the gas-phase end­station (GPES) (Kooser *et al.*, 2020 ▸) of the FinEstBeAMS beamline (Chernenko *et al.*, 2021 ▸). However, future plans include its operation at the photoemission endstation of the FlexPES beamline. The following description includes some details that are specific to the GPES and FinEstBeAMS.

**Figure 1:**
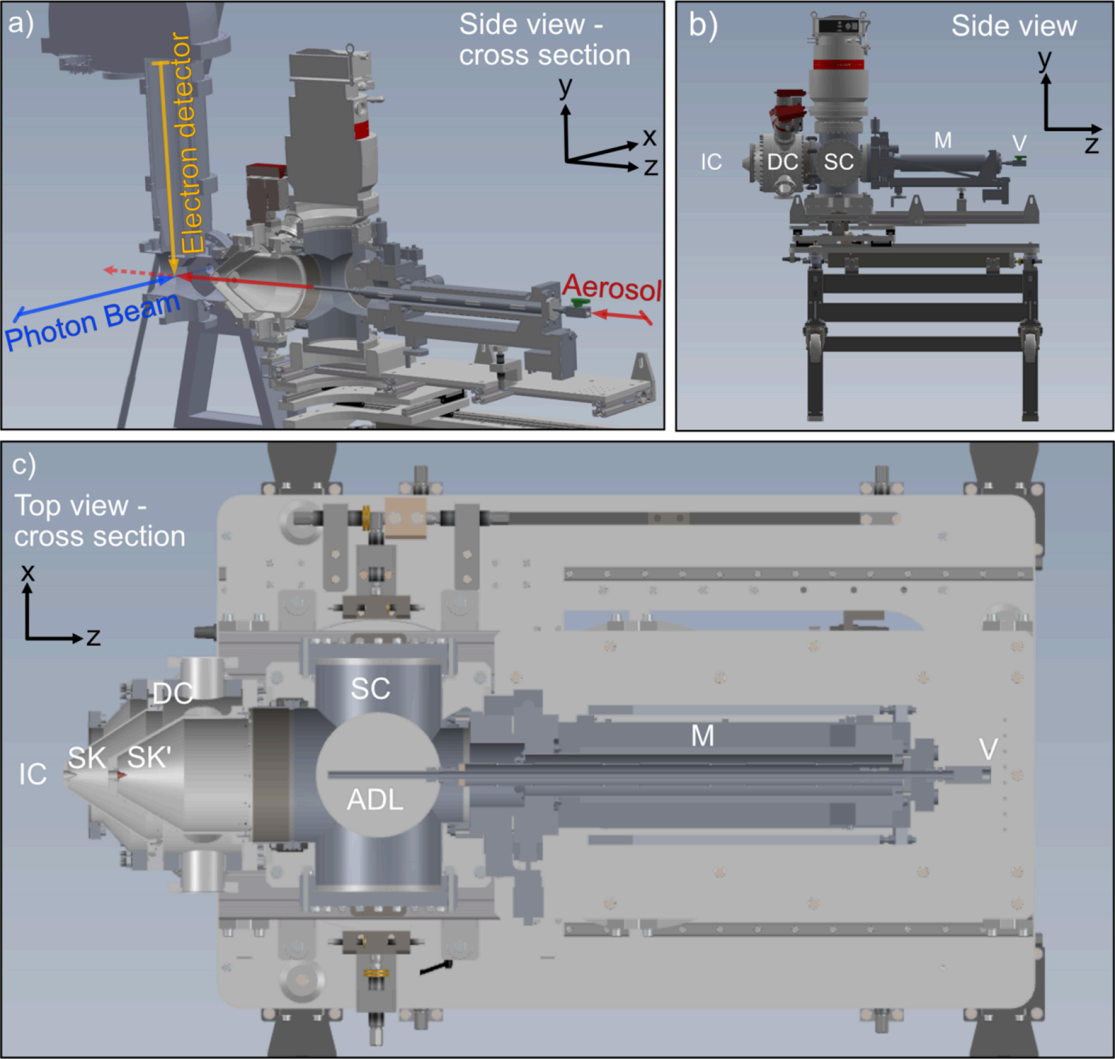
A drawing of the ASDS. (*a*) The ASDS mounted at the FinEstBeAMS beamline, with colored arrows indicating the directions of the photon beam, particle beam and electron detection (the photoelectron spectrometer), and black arrows indicating the coordinate system. (*b*) The ASDS from a side view, placed on the custom-made support table. (*c*) A top view of the ASDS in cross section. The ASDS consists of two vacuum chambers: the source chamber (SC) and the differential pumping chamber (DC). The differential pumping chamber is connected to the interaction chamber (IC, not seen here). Each chamber is separated by a skimmer (SK and SK′). The aerosol enters the ASDS through the valve (V), and travels through the relaxation tube and the ADL that is mounted on the *xyz* manipulator (M).

The salient feature of the ASDS is that it brings aerosols generated by laboratory sources from atmospheric pressure to a vacuum chamber in a continuous flow, enabling in-flight measurements using *e.g.* XPS. The aerosol particles are transmitted through an ADL (Aerodyne PM1) such that, after exiting the ADL, they form a narrow and collimated beam. The defined particle beam intersects the photon beam coming from the (FinEstBeAMS) beamline, just below the end of the electron lens of a SCIENTA R4000 electron analyzer. The SCIENTA R4000 hemispherical electron analyzer measures the kinetic energies of the emitted electrons, Fig. 1 ▸(*a*).

The ASDS is a multi-stage pumping system consisting of two chambers – the source chamber [SC in Figs. 1 ▸(*b*) and 1 ▸(*c*)], which connects to the external aerosol generators via a small 100 µm orifice, and a differential pumping chamber [DC in Figs. 1 ▸(*b*) and 1 ▸(*c*)]. These two chambers are separated by a skimmer [1–2 mm opening diameter, SK′ in Fig. 1 ▸(*c*)]. The differential pumping chamber is separated from the main interaction chamber [IC in Figs. 1 ▸(*b*) and 1 ▸(*c*)] by a second skimmer [0.5–2 mm opening diameter, SK in Fig. 1 ▸(*c*)] that is aligned to ensure overlap of the particle beam with the interaction region of the endstation (GPES), Fig. 1 ▸(*a*).

The source chamber consists of a custom-made six-way cross with four DN200CF ports, a DN200 ISO-K port and a DN160CF port. The top port holds a large 2150 l s^−1^ turbopump, which has sufficient pumping capacity to ensure the removal of a significant portion of the aerosol gas load. This chamber is equipped with DN200CF ports on the front and back sides [as seen in Fig. 1 ▸(*b*)] allowing for the possibility to install one or two further turbopumps. The presently installed Adixen ATH2303H turbopump has proven efficient at maintaining a pressure of ∼1 × 10^−3^ mbar in the source chamber during operation. The right port of the six-way cross [as seen in Figs. 1 ▸(*b*) and 1 ▸(*c*)] is of type DN160CF and is used for mounting an *xyz*-manipulator [M in Figs. 1 ▸(*b*) and 1 ▸(*c*)] that holds the ADL system, allowing adjustment of the lens position to ensure maximum transmission of aerosol particles into the interaction chamber. The left port is of type DN 200 ISO-K and connects to a DN 200 ISO-K–DN 250CF adapter, which holds a custom-made skimmer holder with a skimmer [SK′ in Fig. 1 ▸(*c*)]. This skimmer holder is optional and can even be removed, depending on the experimental needs.

The differential pumping chamber is a custom DN250CF cross with eight DN63CF ports welded at every 45° on its outer cylindrical surface. This chamber can be pumped with a variable number of small turbopumps (for commissioning, three HiPace 80 turbopumps have been used), while the bottom DN63CF flange has been used for additional support. The purpose of the differential pumping chamber is to ensure further removal of background gas so that the pressure in the interaction chamber is sufficiently low (in the 10^−6^ mbar range) to allow operation of the electron spectrometer, as well as ensuring a high signal-to-noise ratio for the measurements. A second skimmer holder with another interchangeable skimmer [SK in Fig. 1 ▸(*c*)] separates the differential pumping chamber from the interaction chamber.

The ASDS is not permanently installed on a specific end­station or beamline and has therefore been designed with mobility and ease of mounting in mind. The differential pumping chamber can be exchanged if a different coupling to another endstation is required. The ASDS rests on a custom-made support table with horizontal and vertical coarse and fine adjustments. In addition, the source chamber sits on a rotatable plate allowing rotational fine adjustments and the chamber rests on rails, which allows easy opening and closing of the connection between the differential pumping chamber and the interaction chamber when maintenance of the skimmers or the ADL is needed. This happens, for instance, when a substantial amount of aerosol particles has been inadvertently deposited on a skimmer, causing it to clog. Unclogging a skimmer requires venting of the ASDS and appropriate cleaning before pumping again. Typically, opening of the source chamber and cleaning of the skimmer causes a downtime of ∼3 h (depending on the extent of cleaning required).

The aerosol particles enter the ASDS through a critical orifice. The critical orifice can be isolated from the source chamber by closing a valve [V in Fig. 1 ▸(*c*)], thus allowing it to be cleaned without breaking vacuum. Downstream of the valve, the aerosol travels through a 0.5’’ outer-diameter 686 mm-long relaxation tube, to which the commercial ADL is connected via ultra-torr Swagelok connections. The relaxation tube is mounted on an *xyz* manipulator such that the ADL position can be adjusted relative to the skimmers, and this arrangement even allows the lens to be fully retracted when maintenance is needed. The length of the relaxation tube does not affect the focusing of the ADL (Lindblad *et al.*, 2013 ▸), and it was specifically chosen so that it would be possible to position the ADL directly in front of the skimmers if required. The pressure in the relaxation tube has been estimated to be 177 Pa (Liu *et al.*, 2007 ▸) when using a 100 µm critical orifice, and the estimated residence time in the relaxation tube is 1.6 s with ∼2% diffusional losses for 100 nm particles (Hinds, 1999 ▸). The residence time can be shortened by shortening the relaxation tube or by using a tube of smaller diameter. This modification can be easily implemented if/when required. For example, in studies where rapid evaporation is expected.

The ADL is an aerosol focusing module that operates with a series of orifices with different opening diameters. The ADL used in this setup is a commercial system (Aerodyne Research Inc.) and its design is based on calculations and evaluations (Liu *et al.*, 1995*a* ▸,*b* ▸, 2007 ▸; Zhang *et al.*, 2002 ▸; Wang, Kruis *et al.*, 2005 ▸; Wang, Gidwani *et al.*, 2005 ▸). At each orifice, the particles are brought closer to the centerline to create a collimated beam, and at the same time the gas molecules expand in transit between two consecutive orifices. The orifice dimensions of the PM1 ADL can be found in the work of Zhang *et al.* (2004 ▸). After the exit of the ADL, the particles are accelerated to high velocity and travel as a collimated beam, whereas the gas molecules expand rapidly and thus can be effectively ‘skimmed’ using the skimmers. The size of the particle beam depends on the distance from the ADL exit, as well as the aerodynamic properties of the particles. A particle beam consisting of small (sub-50 nm) nanoparticles rapidly expands due to diffusion, and heavier particles (above 300 nm, at standard particle density) are overfocused, which also increases the width of the particle beam (Zhang *et al.*, 2004 ▸). The wider the particle beam becomes, the more it is diluted, which will impact the signal of the measurements.

Opposite the ADL, *i.e.* behind the interaction region, a Faraday Cup is mounted for online detection of the aerosol-particle beam. The particles carry a natural charge distribution, where the sum of all charges is a non-zero value. At standard generation parameters, the current from the Faraday Cup is in the picoamps range, and this value is constantly monitored by an electrometer. The Faraday Cup is used for monitoring the status of the particle signal and for alignment purposes.

### 2.2. Operation of the aerosol sample-delivery system

The ASDS has been designed to allow flexibility in terms of the number and size of skimmers that are used in the setup. By removing the skimmer that separates the source and differential pumping chambers, the pressure in the interaction region will be higher; but at the same time such an arrangement allows one to decrease the distance between the exit of the ADL and the interaction region. To avoid damaging the microchannel plates in the electron spectrometer, the maximum allowed pressure in the GPES at FinEstBeAMS is set to 1.3 × 10^−5^ mbar. With this experimental arrangement we observe that it is sufficient to use the ASDS with only one 1 mm skimmer whilst still achieving a suitable pressure in the GPES (low 10^−6^ mbar range). Therefore, for most commissioning experiments presented here, the skimmer separating the source and differential pumping chambers [SK′ in Fig. 1 ▸(*c*)] has been removed. This makes alignment and cleaning much simpler at the expense of higher, but acceptable, pressure in the interaction chamber.

**Figure 2:**
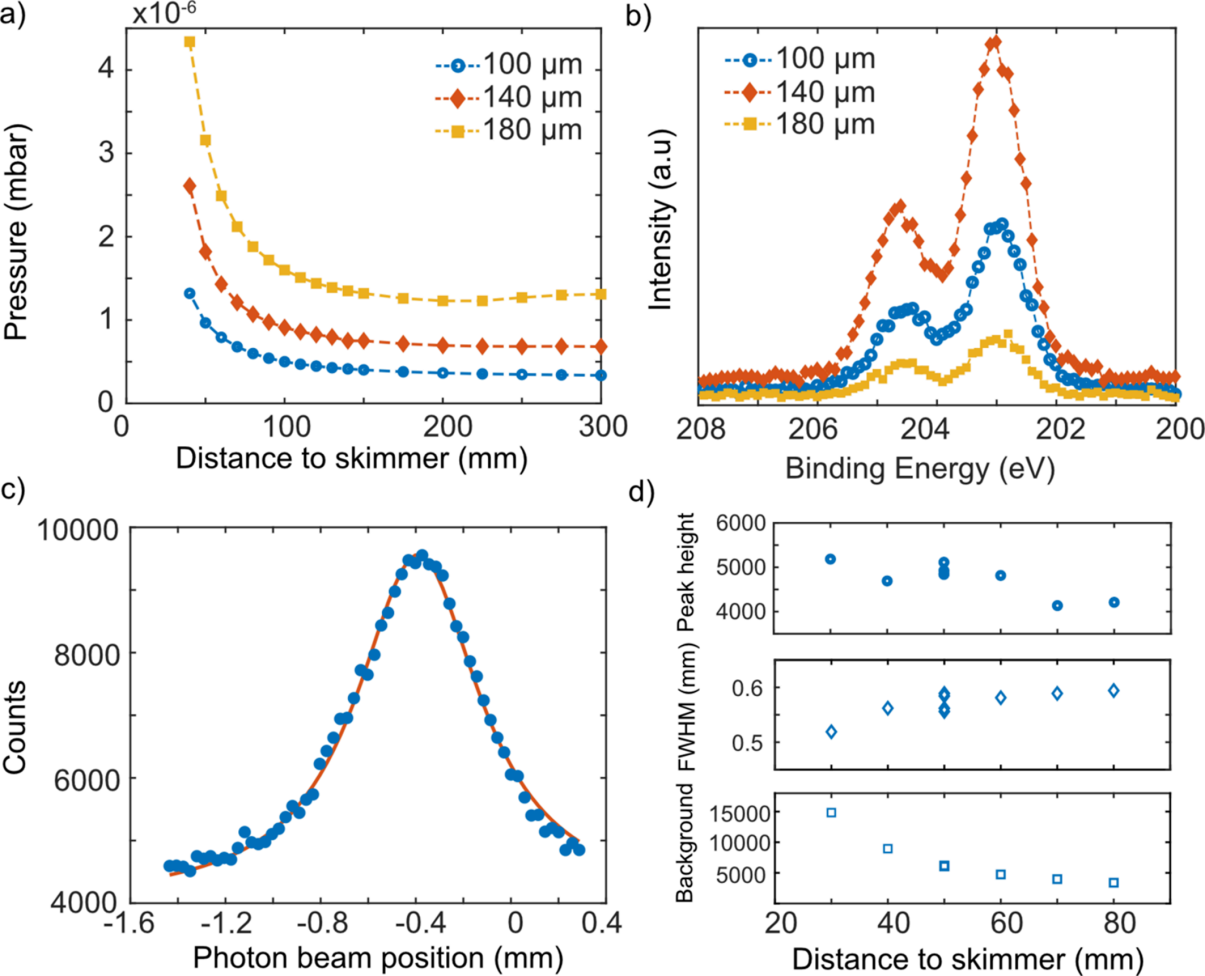
Particle-beam profiles. (*a*) The pressure in the interaction chamber measured as a function of distance between the ADL exit and the skimmer using the *z* motion of the manipulator – measured with orifices of different sizes. (*b*) The Cl 2*p* photoelectron spectrum of NaCl particles measured using orifices of different sizes but with all other conditions unchanged. (*c*) The measured particle-beam profile is fitted with a Lorentzian function and is determined to have an FWHM of ∼0.5 mm. (*d*) The properties of the beam profile (background-subtracted particle-beam peak height, FWHM and background counts) are plotted versus the ADL distance to skimmer. By moving the manipulator closer to the skimmer, FWHM decreases, peak height increases and background counts increase.

The size of the critical orifice, which sits before the valve, governs the aerosol mass flow rate that enters the ASDS and is the first pressure-reducing stage. For most experiments presented here, a 100 µm orifice has been used, but the system has also been tested using a 140 µm and a 180 µm orifice. The measured flow rates through the differently sized orifices were 0.088 (100 µm), 0.149 (140 µm) and 0.265 l min^−1^ (180 µm). A higher mass flow rate will also result in a higher pressure, as shown in Fig. 2 ▸(*a*). The pressure in the interaction chamber is highly dependent on the distance between the exit of the ADL and the skimmer and increases dramatically when the ADL is brought within 50 mm of the skimmer. A larger critical orifice allows a higher gas flow rate into the system, which then yields more particles per unit time through the ADL. However, the higher flow rate may also influence the focusing capability of the ADL (which has been designed for use with a 100 µm critical orifice), and it is therefore not obvious whether a larger orifice opening will result in more particles per unit time in the interaction region or how it will behave for each specific aerosol. This is illustrated in Fig. 2 ▸(*b*), where the intermediately sized orifice gave the strongest signal for nebulized NaCl particles with a salt-solution concentration of 1 g l^−1^.

The photoelectron signal measured by the SCIENTA R4000 analyzer depends on overlap between the photon beam [∼100 × 100 µm at FinEstBeAMS (Chernenko *et al.*, 2021 ▸)] and the aerosol-particle beam below the lens of the electron analyzer. The *xyz* manipulator enables alignment of the particle beam vertically and horizontally but the available range of the aerosol-particle beam is limited to the fixed position of the skimmer relative to the photon beam. The *x* and *y* positions of the skimmer can be adjusted by ±2 mm, but only when the ASDS is open (and thus vented). The vertical position of the photon beam can be adjusted by ∼2 mm at FinEstBeAMS. Fine adjustment of the photon beam is used for finding optimal photon–aerosol-particle overlap, as well as for diagnosing the shape and width of the aerosol-particle beam. The width of the aerosol-particle beam can be determined by measuring the electron signal from a core level (such as Cl 2*p*) of the aerosol particles while moving the photon beam in the vertical direction. Fig. 2 ▸(*c*) shows a typical beam profile for nebulized salt particles with a geometric mean mobility equivalent diameter of ∼100 nm.

By increasing the distance between the exit of the ADL and the skimmers, the pressure in the interaction chamber becomes lower and, thus, less background signal is detected from the gas molecules, but it also results in a wider aerosol-particle beam in the interaction region. In Fig. 2 ▸(*d*), the aerosol-particle beam profile was measured at different distances from the skimmer, by moving the manipulator in the *z* direction over a total distance of 50 mm. From these measurements it can be seen that with increasing distance between the ADL exit and the skimmer, the background-subtracted peak height decreases and, at the same time, the peaks become broader [the full width at half-maximum (FWHM) increases]. By moving the ADL closer to the skimmer, the background counts are observed to increase significantly.

## 3. Commissioning experiments and results

The commissioning experiments presented here have been performed at the FinEstBeAMS beamline in the 1.5 GeV storage ring at the MAX IV Laboratory, Lund, Sweden (Pärna *et al.*, 2017 ▸; Chernenko *et al.*, 2021 ▸; Kooser *et al.*, 2020 ▸). For these measurements, the SCIENTA R4000 electron analyzer was rotated in the vertical direction, and the measurements were performed with photons of linear vertical polarization. In the following sections, results from a wide range of particle types and experiments are presented to demonstrate some capabilities of the ASDS. For all experiments, the kinetic energy of the collected electrons was chosen to have the highest surface sensitivity (∼70 eV), and the investigated core levels were chosen to coincide with the photon energy range where the beamline offers high photon flux and to probe electron orbitals that have high photoionization cross section. The pass energy of the SCIENTA R4000 electron analyzer was set at 100 eV, which together with the use of a 1.5 mm-wide straight slit before the analyzer resulted in a kinetic energy resolution of 375 meV. The photon energy resolution was varied between 100 and 350 meV, depending on the photon energy and intensity of the electron signal. The XPS spectra have been fitted using Gaussian–Lorentzian sum functions with *CasaXPS* (Walton *et al.*, 2010 ▸) version 2.3.23PR1.0 to extract information about the energy positions and width of the peaks.

Since non-supported gas-phase XPS measurements measure the electron energies relative to the vacuum level and not to the Fermi level, an energy shift approximately corresponding to the materials work function is expected compared with the reference values found in the literature. The binding energies were determined relative to the vacuum level after energy calibration using the binding energy of the outermost valence states of N_2_ at 15.58 eV (Dutuit *et al.*, 2013 ▸). During data analysis, we noticed that such energy calibration was not sufficient in some cases, as there existed an additional energy shift related to the kinetic energy of the electrons. The binding-energy scale has been adjusted due to this additional shift, and we estimate the binding energies to be accurate to within ±1 eV in cases where no reference gas molecules were identified in the spectrum.

### 3.1. Inorganic atmospheric aerosols

Inorganic salts are one of the major components of atmospheric aerosol and sea spray is one of the main sources of these salts. Indeed, salt aerosols play a significant role in influencing the Earth’s radiation budget and atmospheric chemical processes, in large part due to their hygroscopicity (*i.e.* tendency to bring moisture from the air into particles) (Zieger *et al.*, 2017 ▸). The new ASDS has been used to investigate the different chemical components of dry salt particles.

The salt aerosol particles were generated with a TSI Constant Output Atomizer Model 3076 with a flow rate of 3 l min^−1^ using dry N_2_ as the carrier gas. The generated droplets were dried with a TSI Diffusion Dryer 3062 and the dry aerosol particles passed through an Ni-63 bipolar neutralizer before entering the ASDS. The flow was split close to the critical orifice of the ASDS to ensure a short distance with low flow rate before the ASDS. Part of the excess flow was guided to a Scanning Mobility Particle Sizer (SMPS, TSI Inc.) for continuously scanning the particle size distributions and the rest of the flow left the system to ventilation. All tubes connecting the different components were mainly stainless steel (6 mm outer diameter) combined with shorter pieces of Tygon tubes, to ensure low losses and impurities. During the initial commissioning tests, we observed that use of flexible conductive silicone tubing caused significant Si surface contaminations on the particles. This has also previously been observed by Yu *et al.* (2009 ▸) and determined to be due to impurities from adsorbed siloxanes.

**Figure 3:**
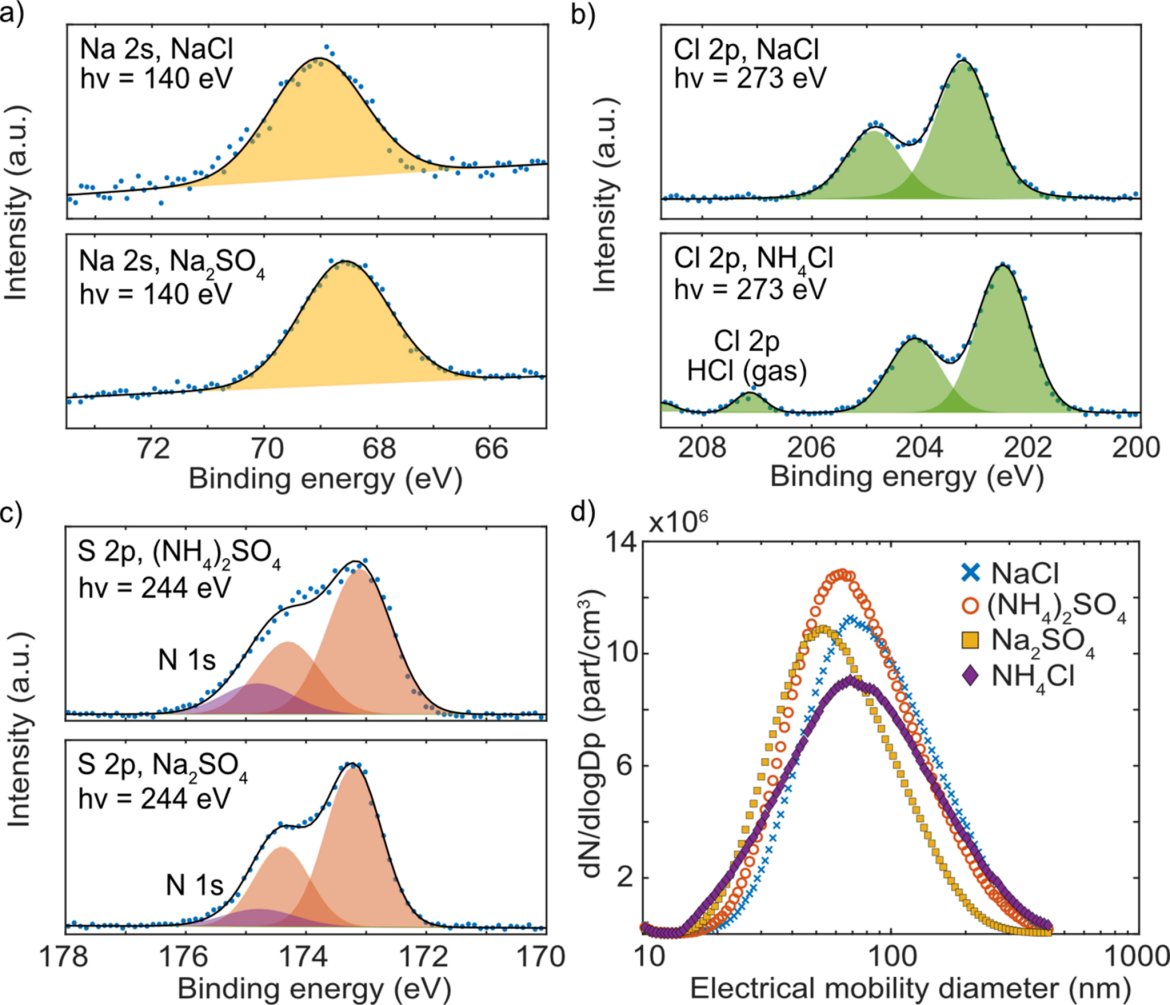
XPS of salt aerosols. (*a*)–(*c*) Na 2*s*, Cl 2*p* and S 2*p* photoelectron spectra have been measured for four different reference salts. The blue dots show the raw data and the black lines are the sum of the fitted individual peaks (shaded profiles). The size distribution measured with SMPS of each salt aerosol is plotted in (*d*).

Different salt solutions [1 g l^−1^ in MilliQ-water of either NaCl, Na_2_SO_4_, (NH_4_)_2_SO_4_ or NH_4_Cl] were prepared, nebulized and dried into solid salt particles, and XPS spectra were measured [Figs. 3 ▸(*a*)–3 ▸(*c*)]. The SMPS scans in Fig. 3 ▸(*d*) show the number concentration and size distribution of each salt aerosol. For these systems, with the following particle concentrations, each XPS spectrum shown in Fig. 3 ▸ was acquired for ∼30 min.

The Na 2*s* peak of NaCl was shifted 0.5 eV relative to the binding energy of the Na 2*s* peak of Na_2_SO_4_, as shown in Fig. 3 ▸(*a*), and both spectra had an FWHM of 1.9 eV, which is wider than that found in the literature for NaCl (1.4–1.5 eV) (Tissot *et al.*, 2016 ▸; Wertheim *et al.*, 1995 ▸). The Cl 2*p* spectra [Fig. 3 ▸(*b*)] showed a characteristic 2:1 intensity ratio between the 2*p*_3/2_ and 2*p*_1/2_ spin–orbit components, and a spin–orbit splitting of 1.6 eV. The Cl 2*p* peaks for NaCl were shifted in binding energy by −0.7 eV relative to NH_4_Cl. For NH_4_Cl, a contribution from HCl molecules in the gas phase was also detected, which will be further described in the next paragraph. Finally, the S 2*p* spectra of Na_2_SO_4_ and (NH_4_)_2_SO_4_ had a 2:1 intensity ratio and a 1.2 eV spin–orbit splitting between the 2*p*_3/2_ and 2*p*_1/2_ components, with FWHMs between 1.1 and 1.2 eV. The two different salts were close in binding energy with a small chemical shift of +0.1 eV for (NH_4_)_2_SO_4_, which is similar to what has been presented earlier on solid samples (Wahlqvist & Shchukarev, 2007 ▸). The S 2*p* spectra were disturbed by a small signal arising from the N 1*s* shake-up satellites of the N_2_ carrier gas, caused by a second-order component in the monochromated radiation (*h*ν = 244 eV, *h*ν_2_ = 488 eV).

The in-flight aerosol-particle measurements allow the simultaneous detection of both gas and particle components. The signal from the gas molecules can be used for energy calibration or to study gas-particle partitioning. During the generation of the NH_4_Cl salt particles [Fig. 3 ▸(*b*)], there was a significant contribution from the HCl (gas) in the spectra. This signal could potentially be used to observe trends in gas formation by determining the ratio between the particle and gas signals.

### 3.2. Secondary organic aerosols

Apart from inorganic salts, organic aerosols are ubiquitous in our atmosphere and are often the dominating component in terms of mass (Turpin *et al.*, 2000 ▸; Kanakidou *et al.*, 2005 ▸). Organic aerosols can be subdivided into primary, *i.e.* emitted as particulate, and secondary organic aerosols (SOAs), which form from gas-phase precursors under atmospheric processing. Atmospheric research has robustly shown SOAs to be more abundant than primary organic aerosols (Jimenez *et al.*, 2009 ▸; Zhang *et al.*, 2007 ▸). However, even though SOA is a major component of atmospheric aerosol, detailed understanding of SOA formation, evolution and physicochemical properties is still lacking (Srivastava *et al.*, 2022 ▸; Fan *et al.*, 2022 ▸).

Flow reactors have emerged as a powerful tool for experimental SOA studies (Zhang *et al.*, 2024 ▸), for example the Organic Coating Unit (OCU) (Keller *et al.*, 2022 ▸). In the OCU, precursor gases are irradiated with UV lamps, either for coating of an introduced seed aerosol or to induce new particle formation. In the current measurements with the ASDS, the OCU was used to produce SOAs from alpha-pinene, an abundant biogenic particle precursor. (NH_4_)_2_SO_4_ was used as the seed particle and was generated as described in Section 3.1, but with a lower concentration of the solution (0.5 g l^−1^) and using dry air as the carrier gas. The salt aerosol flowed at 0.67 l min^−1^ through the OCU chamber.

**Figure 4:**
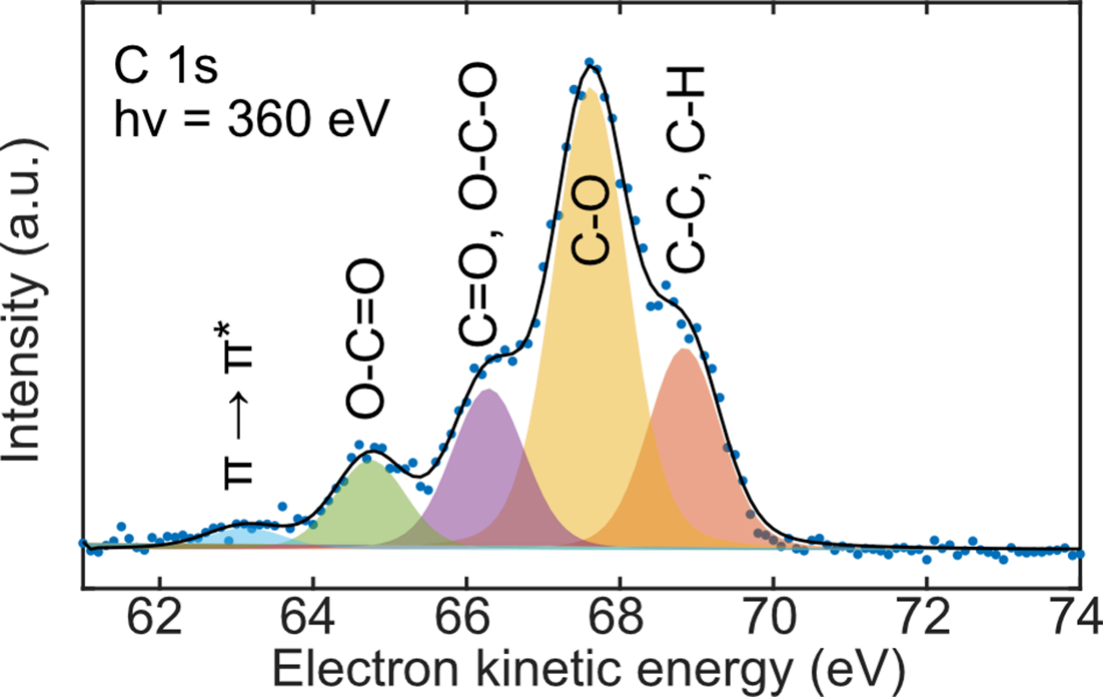
SOA-coated salt particles. The functional groups of C 1*s* are plotted for SOA-coated (NH_4_)_2_SO_4_ particles. The blue dots show the raw data and the black line is the sum of the fitted individual peaks (shaded profiles).

A C 1*s* spectrum was acquired for the SOA-coated salt particles (Fig. 4 ▸). The acquired spectrum is for particle surfaces arising from an approximate hy­droxy radical exposure of 10^8^ molecules cm^−3^ h (Keller *et al.*, 2022 ▸), corresponding to several hours to several days of atmospheric oxidation. SOA consists of a range of functional groups, and to avoid overfitting the spectrum each peak has been separated into five different categories: (i) C—C and C—H, (ii) C—O, (iii) C=O and O—C—O, (iv) O—C=O, and (v) shake-up π → π*. For the SOA measurements, no energy calibrations were performed, and the intensity is plotted against the detected electron kinetic energy. All peaks had an FWHM between 1.1 and 1.2 eV, and all categories, except the shake-up, were separated in binding energy by 1.2–1.5 eV, which is expected according to the literature (Gengenbach *et al.*, 2021 ▸).

### 3.3. Engineered nanoparticles

The large distance between the exit of the ADL and the interaction region, as well as the operational range of the PM1 ADL, makes small single nanoparticles (mobility diameter of <40 nm) extremely difficult to measure with the current setup due to particle-beam broadening caused by high diffusivity. However, studies are possible by, for example, allowing a high concentration of small nanoparticles to collide, coagulate and form agglomerates with aerodynamic mobility diameters optimal for ADL collimation (Martikainen *et al.*, 2021 ▸; Sublemontier *et al.*, 2014 ▸). The ASDS can be used to investigate research questions including surface oxidation and reduction, surface segregation, and core-shell formation of ENPs in the aerosol phase.

#### 3.3.1. Metal oxide nanoparticles

**Figure 5:**
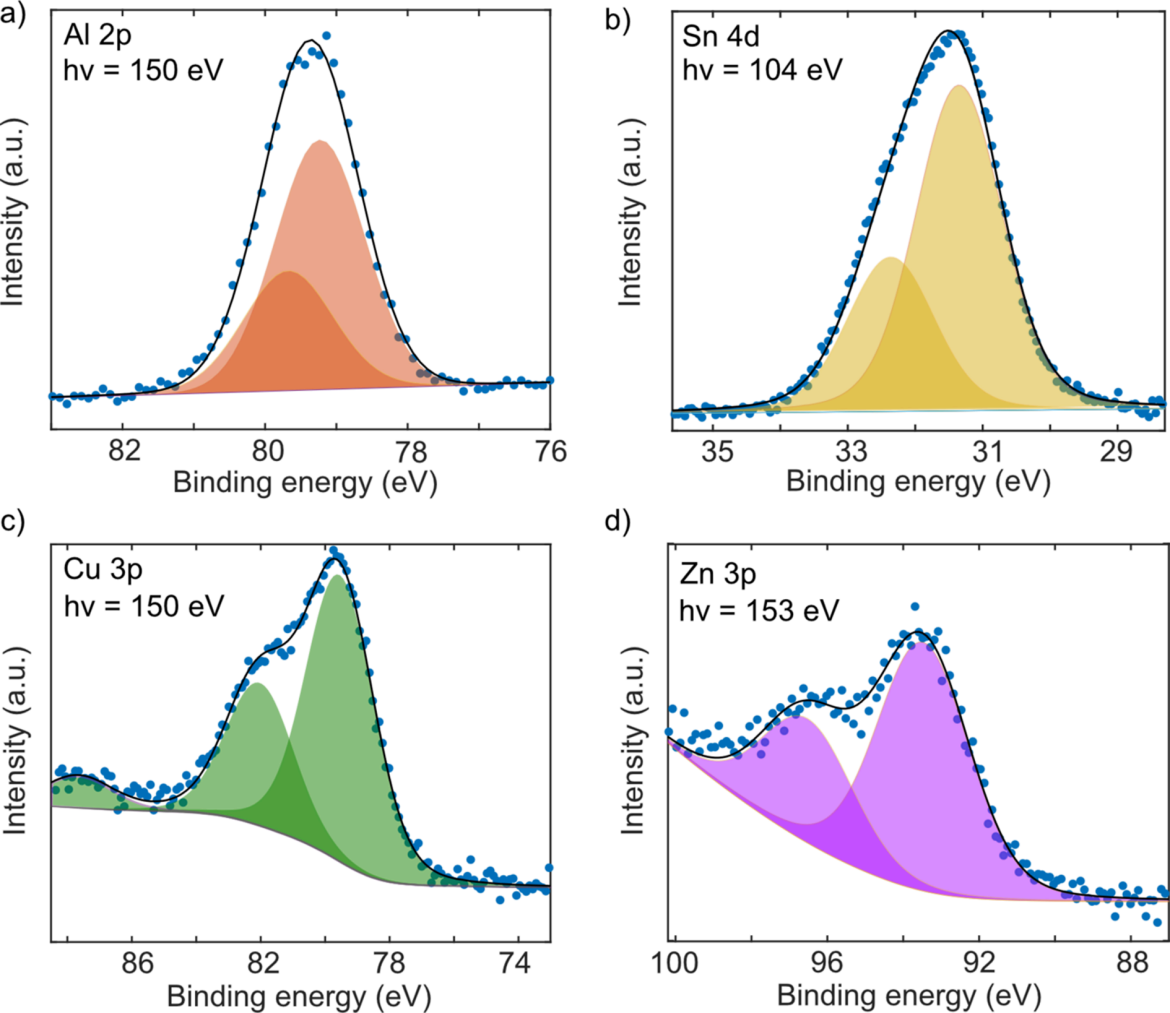
XPS on metal oxide ENPs. Al 2*p*, Sn 4*d*, Cu 3*p* and Zn 3*p* photoelectron spectra have been measured for agglomerates generated from different electrodes. The blue dots show the raw data and the black lines are the sum of the fitted individual peaks (shaded profiles). In all four cases, metal oxide is formed on the surface of the agglomerates. (*a*), (*b*) Al and Sn were generated in a carrier gas composed of N_2_ with 5% H_2_. (*c*), (*d*) Cu and Zn ENPs were generated in N_2_.

ENP agglomerates were generated by spark ablation (Schwyn *et al.*, 1988 ▸; Meuller *et al.*, 2012 ▸; Pfeiffer *et al.*, 2014 ▸). Two opposing electrodes, separated by a gap, were charged to create repeated sparks between the electrodes. The electrode material is ablated by the induced sparks such that the electrodes act as seed material for the nanoparticles being generated. Agglomerates comprising sub-10 nm primary particles of the seed material are promptly formed after each spark (Feng *et al.*, 2016 ▸) and subsequently transported away from the spark region by a carrier gas. Either N_2_ or a gas mixture of N_2_ with 5% H_2_ were used as the carrier gas in these experiments. The latter mixture, with H_2_, was used here to minimize oxidation of the particles in the aerosol phase (Hallberg *et al.*, 2018 ▸). Metal ENPs (Al, Sn, Cu and Zn) were generated using spark ablation and their photoelectron spectra were measured (Fig. 5 ▸).

The Al 2*p* spectrum has been fitted with a spin–orbit splitting of 0.4 eV, with a 2:1 area ratio and a 1.5 eV FWHM. The results are characteristic of the metal oxide (Al_2_O_3_) structure with a wide FWHM (Wagner, 1991 ▸). Any possible signal from Al (0) should be located at 2 eV lower binding energy than the Al_2_O_3_ peaks, and it is not present in the spectrum in Fig. 5 ▸(*a*), showing the absence of metallic Al at the particle surface. The Sn 4*d* spectrum consisted of an Sn (4+) doublet separated by 1 eV, with a 1.5 eV FWHM. No presence of Sn (2+) or Sn (0) was detected in the spectrum; their peaks should be shifted by 0.7 eV and 2 eV, respectively, to lower binding energies relative to those from Sn (4+) (Themlin *et al.*, 1990 ▸; Akgul *et al.*, 2013 ▸). Cu 3*p* has a 2.4 eV spin–orbit splitting and a satellite peak at ∼8 eV higher binding energy than the Cu 3*p*_3/2_ peak (Khalakhan *et al.*, 2021 ▸; Scrocco, 1979 ▸). The binding energies of the observed Cu 3*p* peaks indicate that the surface structure of the Cu ENPs is composed of CuO. Finally, the Zn 3*p* photoelectron lines had an FWHM of 2.8 eV and spin–orbit splitting of 3 eV. The spectrum indicates that the surface is composed of ZnO (Lebugle *et al.*, 1981 ▸).

#### 3.3.2. Size-selected nanoparticles

In the above-described experiments, the full size distributions of the generated particles were measured. For some applications it is essential to study size-selected particles. Therefore, for the ENP agglomerates composed of Al, tests were performed for size-selected particles. Size selection was made in the aerosol phase using a differential mobility analyzer (DMA), which operates by selecting particles based on their electrical mobility. Upon selection, the particles passed through an Ni-63 neutralizer to obtain a bipolar charge distribution. Knowing the particle charge, the particle electrical mobility can be translated into a diameter. For simplicity, the sizes referred to hereafter are the mobility sizes corresponding to singly charged particles. For ENPs with a bipolar charge distribution, most of the charged particles are singly charged, with few multiply charged particles. No neutral particles pass through the DMA, and either positively charged or negatively charged particles are selected. Thus, when implementing size selection with a DMA, significant particle losses are incurred compared with the full size distribution, and thus the signal is expected to decrease substantially.

**Figure 6:**
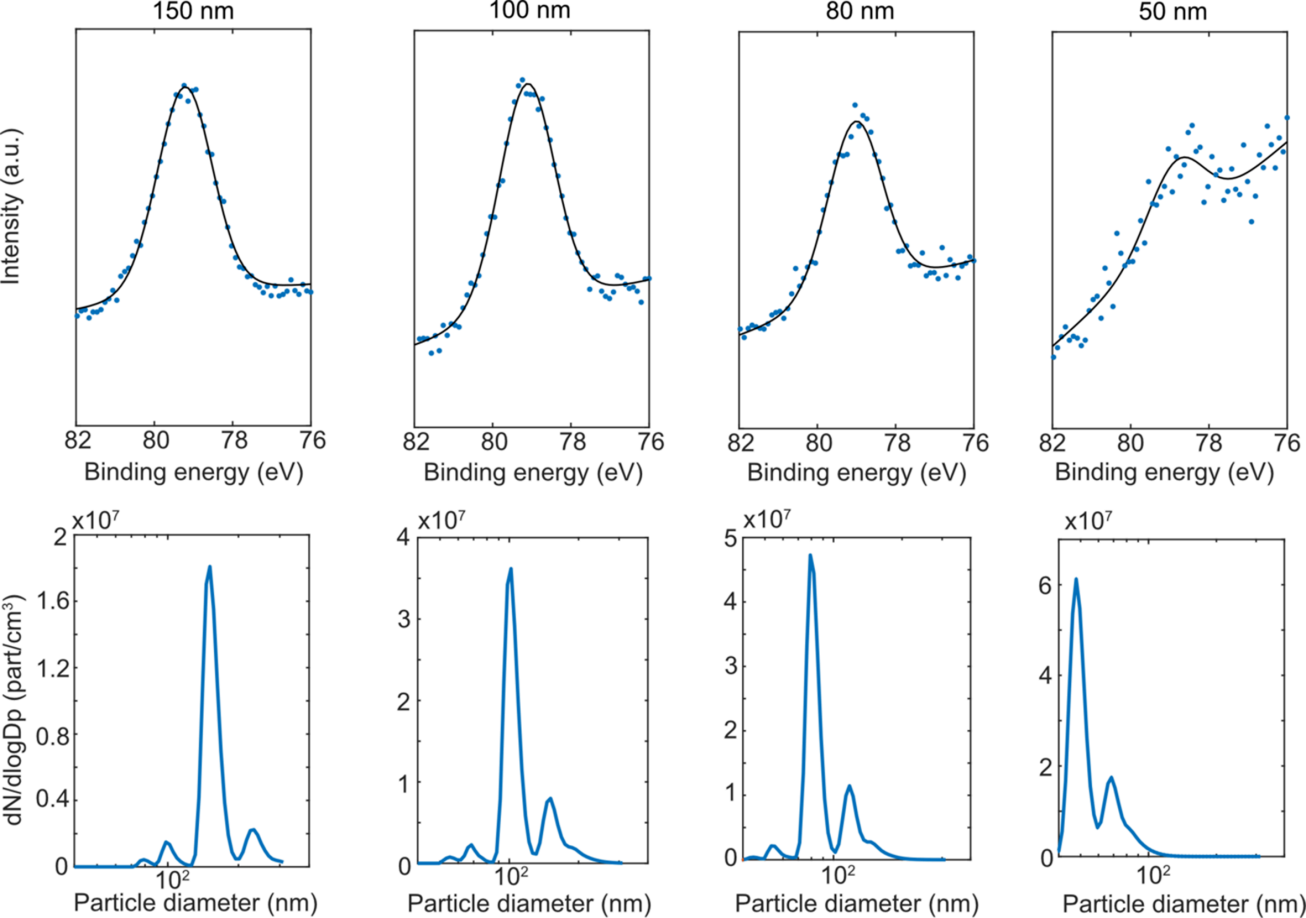
Photoelectron spectra of size-selected ENPs. Top row: Al 2*p* photoelectron spectra from size-selected (150, 100, 80 and 50 nm) ENP agglomerates. The blue dots are raw data and the black line is the peak fit. For small particle diameter, signal becomes low compared with background, which causes a visible slope in the background profile. Bottom row: corresponding SMPS scans for each size-selected system. The main peak corresponds to the size-selected particles and the second peak at higher particle diameter (*i.e.* electrical mobility diameter) corresponds to doubly charged particles. The peak features at lower particle diameters are measurement artefacts.

In the experiments presented here, particles of sizes 150, 100, 80 and 50 nm were selected, and the size distribution of the size-selected particles was simultaneously measured with an SMPS (shown in the bottom row of Fig. 6 ▸). Photoelectron spectra from the size-selected particles (top row, Fig. 6 ▸) were achieved with acceptable signal down to 50 nm. As can be deduced from Fig. 6 ▸, when size selecting with a DMA, multiply charged (larger) particles with the same electrical mobility as the smaller singly charged particles are also likely to be selected. Hence, it is necessary to consider that a fraction of doubly charged particles may always be present when the DMA is used for size selection. The extent of doubly and triply charged particles depends on the original size distribution of the generated particles.

## 4. Conclusions

A versatile ASDS has been developed for in-flight photoelectron spectroscopy studies at the MAX IV Laboratory. The ASDS brings aerosol particles suspended in a gas at atmospheric pressure to vacuum in a continuously renewed, collimated and concentrated particle beam. The delivery system can be operated with various mass flow rates, and consequently a large pressure span in the interaction region. Furthermore, studies of a wide range of different particle types have been demonstrated, including size-selected engineered nanoparticles in the shape of agglomerates, secondary organic aerosols and inorganic aerosol particles. At the same time, the surrounding gas molecules from the aerosols can be studied simultaneously with the particles. The gas molecules can be used for simple energy calibration or to study gas-particle partitioning. Aerosol studies at FinEstBeAMS allow for the collection of photoelectron spectra up to at least the C 1*s* edge. However, using the ASDS at other beamlines will allow for studies at higher photon energies. The ASDS is now available for external users for in-flight studies on chemical surface composition of free unsupported aerosols and nanoparticles.
